# How efficient are metal‐polymer and dual‐metals‐polymer non‐lead radiation shields?

**DOI:** 10.1002/jmrs.733

**Published:** 2023-10-24

**Authors:** Zaker Salehi, Mansour Tayebi Khorami

**Affiliations:** ^1^ Department of Radiation Sciences, School of Paramedical Sciences Yasuj University of Medical Sciences Yasuj Iran

**Keywords:** Lead shields, lead‐free aprons, radiation protection

## Abstract

**Introduction:**

Lead shields are often used to attenuate ionising radiations. However, to make lighter, recyclable and more efficient shields compared to lead, combinations of new metallic compounds together with polymer, for example, flexible polyvinyl chloride (PVC) have been developed recently. In this study, the capabilities of non‐lead radiation shields made of one or two metallic compounds and polymer were evaluated.

**Methods:**

Monte Carlo (MC)‐based BEAMnrc code was used to build a functional model based on a Philips X‐ray machine in the range of radiographic energies. The MC model was then verified by IPEM Report 78 as a standardised global reference. The MC model was then used to evaluate the efficiency of non‐lead‐based garments made of metallic compound and polymer (MCP) including BaSO_4_‐PVC, Bi_2_O_3_‐PVC, Sn‐PVC and W‐PVC, as well as dual‐metallic compounds and polymer (DMCP) including Bi_2_O_3_‐BaSO_4_‐PVC, Bi_2_O_3_‐Sn‐PVC, W‐Sn‐PVC and W‐BaSO_4_‐PVC. The absorbed doses were determined at the surface of a water phantom and compared directly with the doses obtained for 0.5 mm pure lead (Pb).

**Results:**

Bi_2_O_3_‐BaSO_4_‐PVC and W‐BaSO_4_‐PVC were found to be efficient shields for most of the energies. In addition to the above radiation shields, Bi_2_O_3_‐Sn‐PVC was also found to be effective for the spectrum of 60 keV. Bi_2_O_3_‐BaSO_4_‐PVC as a non‐lead dual metals‐PVC shield was shown to be more efficient than pure lead in diagnostic X‐ray range.

**Conclusion:**

Combination of two metals‐PVC, a low atomic number (Z) metal together with a high atomic number metal, and also single‐metal‐PVC shields were shown to be efficient enough to apply as radiation protection shields instead of lead‐based garments.

## Graphical abstract

Replacing lead radiation aprons with lead‐free aprons is a challenge in radiation protection. In this study, the attenuation capability of non‐lead radiation shields made of one or two metallic compounds together with polymer was evaluated. The results showed that some dual‐metals‐polymer compounds such as Bi_2_O_3_‐BaSO_4_‐PVC are capable of reducing the radiation dose significantly better than pure Pb and some metal‐polymer compounds have adequate protection in diagnostic energies.

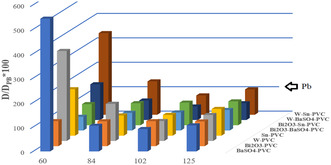

## Introduction

The use of ionising radiation in medical imaging helps to diagnose diseases within the human body. Medical imaging modalities with ionising radiation, such as X‐ray radiation, increase the collective dose of the population due to the increasing rate of examinations.^1^ One of the radiation protection methods is the use of protective aprons, which are used in diagnostic X‐ray imaging, angiography and intervention procedures.^2^, ^3^, ^4^, ^5^ The use of lead (Pb) in protective aprons for the purpose of radiation protection has been common selection in the past years. Two important factors for choosing aprons are the weight and the protection provided by these shields. These aprons should have radiation attenuation equivalent to 0.25, 0.5 and 1 mm of lead.^6^, ^7^, ^8^, ^9^ In order to create flexibility in the aprons, silicone rubber or polymer materials, such as flexible PVC, are applied.^7^, ^10^, ^11^ For the purpose of weight reduction and to eliminate the lead health hazards and environmental pollution, the replacement of other materials instead of lead in radiation protection aprons has been considered.^12^ The materials include tungsten (W), Bismuth oxide (Bi_2_O_3_), Barium sulphate (BaSO_4_) and tin (Sn) which increase the radiation attenuation at a certain range of energies due to the K‐edge absorption and are commonly used in the manufacture of the radiation protection aprons.^10^, ^13^, ^14^ The wide range of X‐ray energies cause difficulty in selecting efficient shielding materials according to K‐edge absorption.^3^, ^11^, ^15^ Because of the heavy weight, aprons put a lot of pressure on the vertebral column and intervertebral discs. Hence, reducing the weight of the apron is necessary for radiation workers that have to use them for relatively long time periods.^16^ On the other hand, weight loss of shields should not result in increasing the radiation exposure of radiation workers. For this reason, investigating the radiation absorption properties of non‐lead shielding has been the aim of many studies. The aim of this study was to evaluate the effectiveness of non‐lead materials applied in radiation shielding garments.

## Methods

BEAMnrc, a Monte Carlo (MC) code, version 4.2.3.1 was used to derive the energy spectrums for 60, 84, 102 and 125 kVp X‐rays which are common to clinical radiological applications. The simulation was based on a Philips X‐ray machine, model SRO/ROT 350. The software was installed on a Linux, Ubuntu 18.04.1 LTS, machine with Intel Core (TM) i3‐9100F, 3.6GHz*4. All the spectrums were obtained using 500 million particles and a 2.5 mm aluminium filter as a total equivalent filter. The obtained spectrums were then compared with IPEM report 78 to evaluate the accuracy of the Monte Carlo model applied for the current study. This report of IPEM (The Institute of Physics and Engineering in Medicine) provides a collection of diagnostic X‐ray spectra together with linear attenuation coefficients for commonly used materials in X‐ray machines.^17^

Then, to fabricate the digitalised radiation shields to be used in the simulation, metallic compound‐polymer (MCP) shields and dual‐metallic compounds‐polymer (DMCP) shields were considered. For the MCP shields, 75% metal compound and 25% polymer were considered, and for DMCP shields, 37.5% of the first metal compound along with 37.5% of the second metal compound together with 25% of polymer were used. MCP shields were made with BaSO_4_, Bi_2_O_3_, Sn and W along with polymer (BaSO_4_‐PVC, Bi_2_O_3_‐PVC, Sn‐PVC, W‐PVC) and DMCP shields were made of Bi_2_O_3_‐BaSO_4_, Bi_2_O_3_‐Sn, W‐Sn and W‐BaSO_4_ along with polymer. 0.5 mm pure Pb was used as the reference shield. The thickness of the radiation shields was calculated such that the grammage (the mass per unit area expressed in terms of g/cm^2^) of all of the radiation shield equals to the grammage of 0.5 mm Pb as the reference shield (See Table 1). The source to shield distance was considered as 100 cm, and dose measurements were performed, just behind the shield, on the surface of a water phantom. The ratio of dose values obtained for all shields to the reference shield (pure Pb) at the surface of the water phantom was obtained. Also, the ratio of dose for various depths of water phantom to the dose of the beam at the absence of shields was determined.

**Table 1:** The dual‐metals‐PVC shields and single‐metal‐PVC shields materials characteristics. All compounds have the same grammage (g/cm^2^) which equals to the grammage of 0.5 mm pure Pb.

Mixture	Weight percent (%)										Density (g/cm^3^)	Thickness (mm)	K‐edge (keV)
	Pb	W	Sn	Ba	Bi	O	S	Cl	C	H			
Pb	100	0	0	0	0	0	0	0	0	0	11.34	0.5	88
Sn‐PVC	0	0	75	0	0	1.64	0	8.51	13.15	1.7	3.38	1.68	29.2 (Sn)
W‐PVC	0	75	0	0	0	1.64	0	8.51	13.15	1.7	4.32	1.31	69.5 (W)
BaSO_4_‐PVC	0	0	0	44.13	0	22.2	10.3	8.51	13.15	1.7	2.78	2.04	37.4 (Ba)
Bi_2_O_3_‐PVC	0	0	0	0	67.27	9.36	0	8.51	13.15	1.7	3.61	1.57	90.5 (Bi)
Bi_2_O_3_‐BaSO_4_‐PVC	0	0	0	22.06	33.64	15.78	5.15	8.51	13.15	1.7	3.14	1.81	90.5 (Bi) & 37.4 (Ba)
Bi_2_O_3_‐Sn‐PVC	0	0	37.5	0	33.64	5.5	0	8.51	13.15	1.7	3.49	1.62	90.5 (Bi) & 29.2 (Sn)
W‐BaSO_4_‐PVC	0	37.5	0	22.06	0	11.92	5.15	8.51	13.15	1.7	3.39	1.67	69.5 (W) & 37.4 (Ba)
W‐Sn‐PVC	0	37.5	37.5	0	0	1.64	0	8.51	13.15	1.7	3.79	1.49	69.5 (W) & 29.2 (Sn)

Ba, Barium; Bi, Bismuth; C, Carbon; Cl, Chlorine; H, Hydrogen; O, Oxygen; Pb, Lead; S, Sulfur; Sn, Tin;W, Tungsten.

## Results

**Figure 1:**
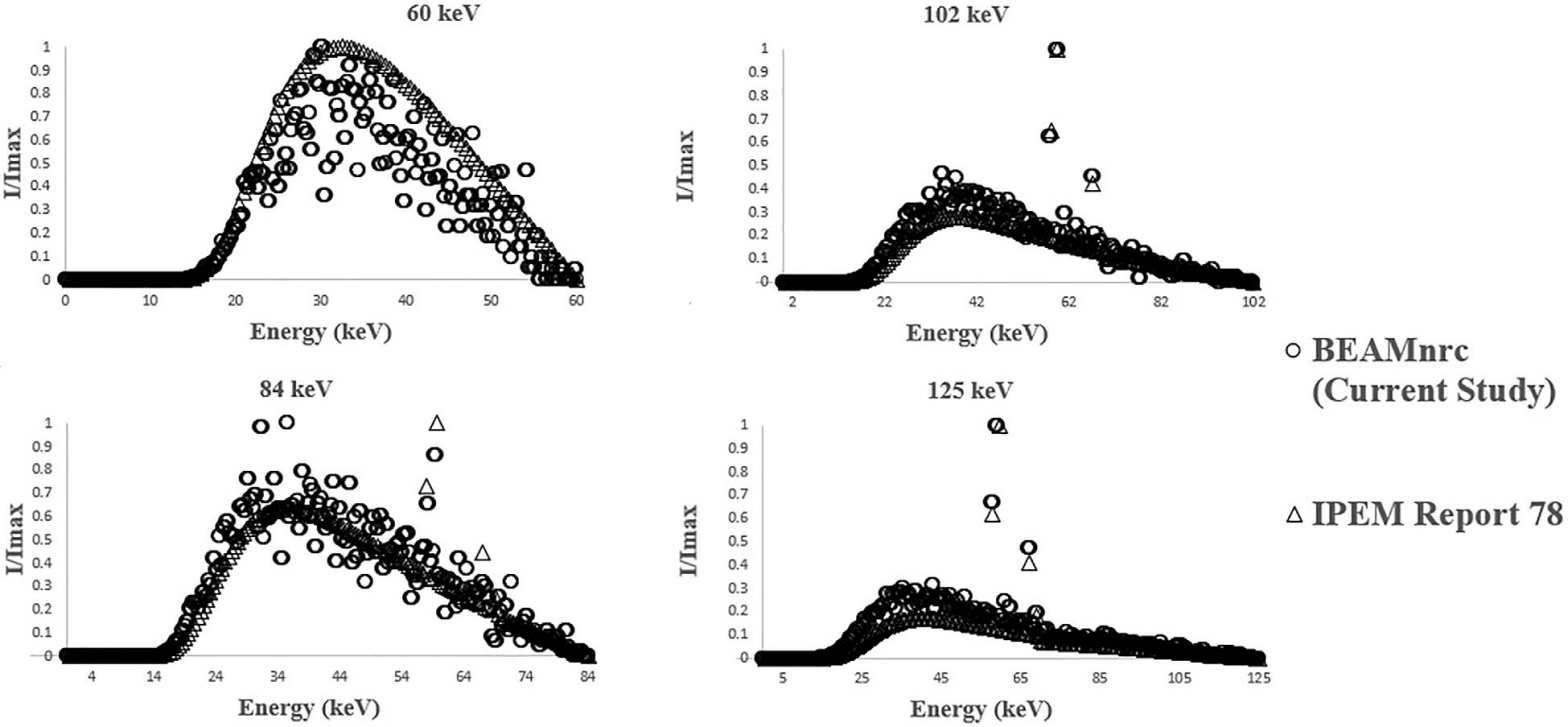
X‐ray Spectrums of four common diagnostic energies built by BEAMnrc for 500 million particles based on a Philips x‐ray machine (at the tube potential of 60, 84, 102 and 125 kVp). IPEM Report 78 was used to verify the Monte Carlo model of current study.

The comparison between the X‐ray spectrums determined by IPEM Report 78 and MC model (current study) are demonstrated in Figure 1 for four diagnostic energies. As it is shown, our results are in good agreement with IPEM results and as energy is increased the agreement is getting better. Furthermore, for numerical comparison between the spectrums, the first half value layer (1st HVL) values derived from both methods were compared to each other. The discrepancies between the HVLs for IPEM data and MC model range from 6.8% for 60 kVp to 3.6% for 125 kVp which means the discrepancy is smaller as the beam is hardened.

**Figure 2:**
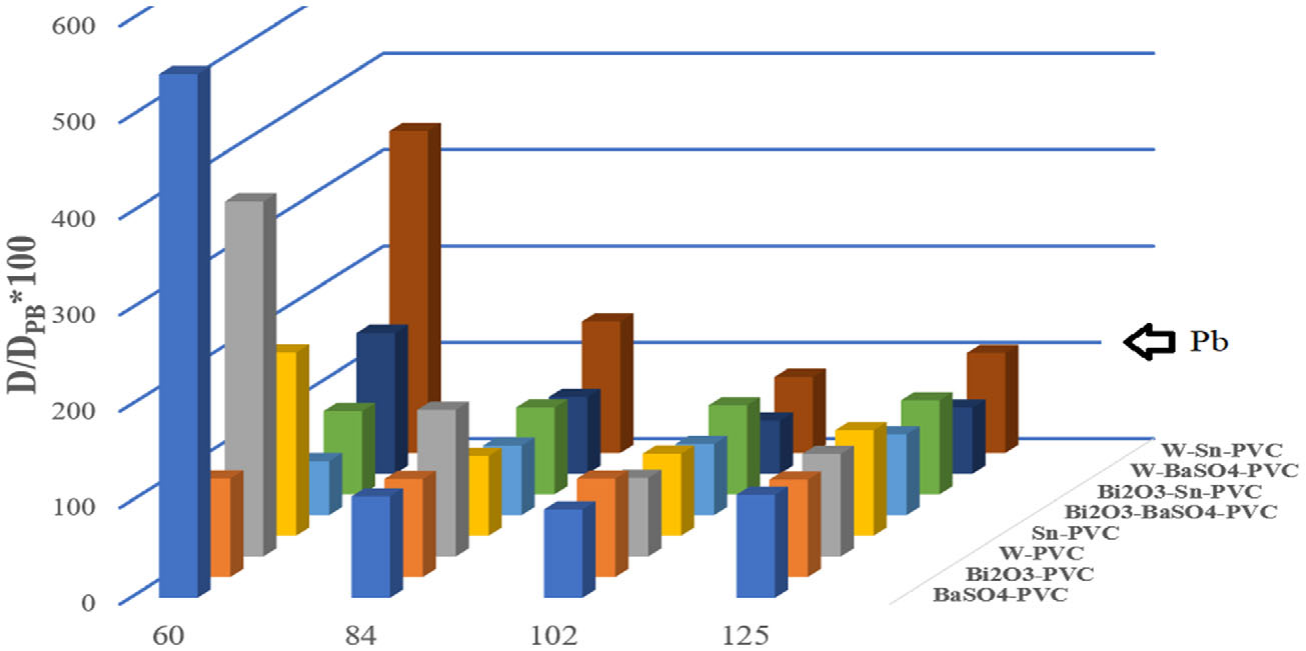
Comparison between the surface dose of 0.5 mm pure lead (as the reference shield) with the metal‐PVC and dual‐metals‐PVC shields for diagnostic X‐rays (60, 84, 102 and 125 kVp).

Figure 2, compares the ratio of doses after lead‐free shields to 0.5 mm pure lead in different energies. According to Figure 2, for the spectrum of 60 kVp, Bi_2_O_3_‐BaSO_4_‐PVC and Bi_2_O_3_‐Sn‐PVC have lower entrance doses than the reference shield and Bi_2_O_3_‐PVC has the same dose as the reference shield. In the spectrum of 84 kVp, Sn‐PVC, Bi_2_O_3_‐BaSO_4_‐PVC, Bi_2_O_3_‐Sn‐PVC and W‐BaSO_4_‐PVC have lower entrance doses than the reference shield. Moreover, BaSO_4_‐PVC and Bi_2_O_3_‐PVC have the same doses as the reference shield. In the spectrum of 102 kVp, all shields except Bi_2_O_3_‐PVC have a lower surface dose than the reference shield. Furthermore, in the spectrum of 125 kVp, all four single metal shields have the same dose as Pb and all dual metal shields except W‐Sn‐PVC have a lower surface dose than the reference shield. Table 2 indicates the ratio of doses with radiation shields at different depths of the water phantom to the maximum dose without the shield at the surface of a water phantom. As shown in Table 2, W‐BaSO_4_‐PVC and Bi_2_O_3_‐BaSO_4_‐PVC shields showed better capability of attenuation compared to other shields in different diagnostic energies.

**Table 2:** %*D*/*D*_max_ versus depth for all eight shields and four energies, the ratio of doses with shields at different depths of water phantom to the maximum dose without shield at the surface of water phantom.

Spectrum (kVp)	Depth (cm)	% (*D*/*D*_max_)							
		Metallic compound polymer (MCP) shields				Dual metallic compound polymer (DMCP) shields			
		Bi_2_O_3_‐PVC	BaSO_4_‐PVC	Sn‐PVC	W‐PVC	Bi_2_O_3_‐BaSO_4_‐PVC	Bi_2_O_3_‐Sn‐PVC	W‐BaSO_4_‐PVC	W‐Sn‐PVC
60	0	0.21	1.12	0.39	0.76	0.11	0.18	0.30	0.69
	2.5	0.14	0.69	0.17	0.53	0.07	0.12	0.19	0.47
	5	0.09	0.39	0.08	0.35	0.05	0.08	0.13	0.31
	7.5	0.06	0.22	0.03	0.22	0.03	0.05	0.08	0.20
84	0	2.45	2.53	1.99	3.65	1.73	2.17	1.92	3.28
	2.5	1.88	1.63	1.24	2.84	1.33	1.73	1.49	2.67
	5	1.40	1.00	0.85	2.03	1.00	1.30	1.01	1.88
	7.5	1.02	0.61	0.55	1.39	0.74	0.91	0.70	1.29
102	0	5.76	5.16	4.79	4.59	4.15	5.19	3.10	4.45
	2.5	3.84	3.18	3.12	3.65	3.08	3.50	2.22	3.41
	5	2.84	1.95	2.05	2.57	2.19	2.72	1.64	2.45
	7.5	1.95	1.30	1.48	1.84	1.46	1.86	1.08	1.73
125	0	6.85	7.23	7.42	7.19	5.68	6.61	4.64	7.03
	2.5	5.32	5.30	5.74	5.59	4.32	4.99	3.83	5.32
	5	3.91	3.80	4.06	3.86	3.11	3.58	2.74	3.64
	7.5	2.79	2.57	2.97	2.71	2.39	2.55	1.88	2.54

## Discussion

Figure 2 shows that Bi_2_O_3_‐PVC is the most similar shield to the reference shield (Pb), which can be due to the proximity of the K absorption edge of bismuth to the K absorption edge of lead. In general, it can be concluded that almost all DMCP shields have better efficiency in all diagnostic energies compared to Pb. Figure 2 and Table 2 show *D*/*D*_(pure Pb)_ and *D*/*D*_max_ for all energies used in this study. As it can be seen, combined shields have better protective results than pure Pb or single‐metal shields in range of low‐energy X‐rays. In this research, the common compounds in lead‐free aprons have been investigated in terms of radiation protection efficiency. Since a great proportion of radiation attenuation in protective aprons is provided by photoelectric absorption (the elements with K absorption edge in the energy range of diagnostic radiology). Since most primary rays are absorbed by photoelectrons due to photoelectric effects, and secondary rays (scattering rays) are generated consequently, the surface dose increases right behind the shields.^15^, ^18^, ^19^ Therefore, the dose ratio at the surface of water phantom right after the radiation shields has been investigated (Fig. 2). In the current study, BEAMnrc and DOSXYZnrc were used to obtain X‐ray spectrums and absorbed doses. These codes are used to simulate the electron‐photon transport for radiation dose calculations.^20^, ^21^, ^22^ The IPEM reports provide standard data to validate the radiation spectrum.^22^ In order to check the validity of the results of this research, the spectrums produced in four diagnostic energies were directly compared with IPEM Report 78. As shown in Figure 1, the spectrums at maximum energies of 60, 84, 102 and 125 keV have good agreement with IPEM data, but at spectrum of 60 kVp, there are some deviations in the results, which can be due to the greater accuracy of IPEM at higher energies. The broad beam geometry was used for this study, which is more suitable for examining protective aprons because it measures the primary and secondary rays that are effective in the absorbed dose of radiation workers. Considering the different density of materials used in the shields, in this research, the same grammage (the basic weight expressed in terms of gram per square centimetre) of all shields have been applied. Due to the selection of different thicknesses based on the density of each shield, all shields will have the same weight and therefore the effects of density and weight will not affect the results. In the research conducted by Moonkum et al.,^10^ the percentage of radiation absorption by silicone rubber shields containing Bi_2_O_3_ and BaSO_4_ was measured at two energies of 80 and 120 keV, which showed a higher absorption percentage in the shield containing barium sulphate at both energies. It should be noted that in Moonkum's research, radiation shields with the same thickness were used. Kim et al.^1^ showed that in the same thickness, yarns' shields containing Bi_2_O_3_ and BaSO_4_ have good protective performance, and the yarns containing bismuth are efficient in effective energies of 25.2 to 57.3 keV. Schlattl et al.^18^ investigated the effectiveness of tin, tin‐bismuth and lead shields in the energy spectrum of 60 kVp and showed that the surface dose is the highest while using the tin shield, and it will be lowest using the lead shield. As can be seen in Figure 2, the relative dose of tin containing shield is higher than bismuth‐tin containing shield. But the surface dose of bismuth‐tin is lower than that of lead, and this disparity can be caused by the difference in the percentage of bismuth and tin (used in Schlattl research). It should be noted that the bismuth‐tin shield in Schlattl research contained 20% of bismuth and 80% of tin, while in current research, the equal weight percentage of tin and bismuth were used. Kazempour et al.^13^ compared several shielding materials and introduced some with better efficiency. It should be noted that they considered all shields with the same thickness and different weights. while in this study, the shields with the same weight were studied. It is important to mention that in this research, the grammage (the mass per unit area in terms of g/cm^2^) of shields are considered the same so that in the absence of confounding factors, the efficiency of non‐lead shields can be evaluated compared to pure lead. By choosing a non‐lead shield with high efficiency (in accordance with current study) and less thickness, lighter garment can be produced as well.

## Conclusion

Monte Carlo simulation (MC) offers an alternative to calculate the absorbed dose during radiography. To verify the MC model of an X‐ray machine, the spectra results have been compared with IPEM Report 78. In summary, the present study indicates that the MC model applied to simulate the X‐ray beams can speed up spectra generation without sacrificing accuracy. The MC models in current study were then used to determine the radiation absorbed doses immediately behind various shields of non‐lead materials exposed to the broad beam of 60, 84, 102 and 125 keV. The results showed that for intermediate and high radiography energies (such as 84, 102 and 125 keV), the combination of a low Z together with a high Z material together with PVC (such as Bi_2_O_3_‐BaSO_4_‐PVC) is capable of reducing the surface dose significantly better than pure Pb. It is also concluded that, for low radiography energy such as 60 keV, although Bi_2_O_3_‐BaSO_4_‐PVC is capable to be used as an effective shield, However, Bi_2_O_3_‐Sn‐PVC showed better efficiency relatively.

## Conflict of Interest

The authors declare no conflict of interest.

## Ethics Statement

The research has institutional ethics approval.

## Data Availability

The article describes entirely theoretical research.
